# Beyond composite scores in chronotype assessment: item-level predictive patterns in the Morningness–Eveningness Questionnaire

**DOI:** 10.1038/s41598-026-54301-w

**Published:** 2026-06-04

**Authors:** Yannick A. Metzler, Hannah M. Schade, Michael A. Nitsche, Edmund Wascher, Stephan Getzmann, Patrick D. Gajewski, Lorena Melo

**Affiliations:** 1 Department of Ergonomics, Leibniz Research Centre for Working Environment and Human Factors (IfADo), Dortmund, Germany; 2 Department of Psychology and Neurosciences, Leibniz Research Centre for Working Environment and Human Factors (IfADo), Ardeystraße 67, 44139 Dortmund, Germany; 3 German Center for Mental Health (DZPG), Partner Site Bochum/Marburg, Bochum, Germany; 4 University Clinic of Psychiatry and Psychotherapy, Protestant Hospital of Bethel Foundation, University Hospital OWL, Bielefeld University, Bielefeld, Germany; 5 SensoriMotor Laboratory, Department of Ophthalmology, Fondation Asile des Aveugles, Jules-Gonin Eye Hospital, University of Lausanne, Lausanne, Switzerland

**Keywords:** Chronotype, Machine learning, Morningness–Eveningness Questionnaire, MEQ, Circadian preference, Psychological assessment, Item response pattern, Health care, Medical research, Psychology, Psychology

## Abstract

Chronotype represents individual differences in circadian preferences that influence sleep-wake patterns, cognitive performance, and clinical outcomes across the lifespan. Given its growing relevance for clinical research and application, efficient and reliable chronotype assessment is essential. However, widely used tools like the Morningness–Eveningness-Questionnaire (MEQ) rely on composite scores, despite methodological concerns about multidimensionality and unequal item contributions. Addressing this limitation, the current study applied machine learning techniques to enhance theoretical understanding and empirical precision in chronotype assessment. Using the German MEQ in the Dortmund Vital Study (ClinicalTrials.gov NCT05155397), a prospective cohort study on healthy cognitive aging, we identified item-level predictive hierarchies and response patterns distinguishing morning, neutral, and evening types. Item 19 (“Which chronotype do you think you are?“) demonstrated exceptional predictive utility, showing threefold greater importance than any other item. This metacognitive self-assessment appears to capture relatively accurate chronotype identification rooted in lived experience. Chronotype-specific analyses revealed distinct predictive patterns across types, each relying on different item combinations for optimal classification. Partial dependence analysis identified non-linear response patterns including sigmoid curves, threshold effects, and plateau regions. A six-item combination achieved robust overall classification, potentially reducing assessment burden by 70%. Overall, this research advances chronotype assessment by uncovering non-linear and heterogeneous classification mechanisms to circadian preference. The findings provide evidence-based foundations for developing abbreviated screening tools for primary care consultations, large-scale epidemiological studies, and clinical contexts. An open-access R tutorial ensures reproducibility and facilitates adaptation across diverse populations and assessment instruments, supporting widespread implementation in research and practice.

**Supplementary Information:**

The online version contains supplementary material available at 10.1038/s41598-026-54301-w.

## Introduction

Chronotype refers to interindividual differences in preferred timing of sleep, activity, and cognitive performance, shaped by circadian, behavioural, and environmental influences^1–3^. Increasing evidence supports its clinical relevance, with evening types showing higher vulnerability to mood and anxiety disorders, and emerging evidence suggesting chronotype-related differences in pharmacological responses^4,5^.

Yet, current assessment tools remain limited. The *Morningness–Eveningness Questionnaire* (MEQ; 6), one of the most widely used measures, employs a composite scoring system that equally weighs all 19 items, despite substantial methodological concerns^7^. This assumption of equal contribution may be fundamentally problematic: chronotype is not necessarily a latent construct in the classical psychometric sense, but rather a typological classification scheme reflecting directly observable circadian preferences with clear physiological correlates^8^. Recent factor-analytic work further demonstrates that the MEQ measures multiple distinguishable constructs rather than a unidimensional trait^9^. But even when considering chronotype as a latent construct, acceptable psychometric properties do not guarantee predictive utility for classification purposes.

Traditional psychometrics prioritize population-level parameter estimation and assume linear relationships between items and latent constructs. Machine learning classification methods, by contrast, prioritize individual-level prediction accuracy and can capture complex, non-linear relationships without requiring a priori assumptions about underlying structures^10,11^. This distinction is particularly relevant for chronotype assessment, where the objective is accurate classification into behavioral phenotypes rather than estimation of an abstract latent trait. Individual circadian preferences involve complex interactions between biological, environmental, and behavioral factors that linear models cannot adequately capture. This distinction matters because chronotype represents direct typologies including observable circadian preferences with clear physiological correlates, rather than an abstract psychological construct that must be inferred indirectly. When the goal is accurate classification into meaningful behavioral categories, the question shifts from “do items measure the same underlying dimension?” to “which items best pre-dict real-world chronotype distinctions?”

Drawing on machine learning techniques, this study examines which MEQ items have significant predictive power for chronotype classification. Specifically, we investigate^1^: which items best predict morning, evening, and neutral chronotypes and whether each type depends on distinct item subsets^2^; what novel insights about chronotype assessment emerge from machine learning approaches compared to existing psychometric analyses; and^3^ what item-specific response patterns differentiate chronotypes and what these patterns reveal about underlying differences between types. By identifying item-level predictive utility hierarchies and chronotype-specific response patterns, this work provides foundations for empirically advancing chronotype assessment and developing abbreviated screening tools while maintaining classification accuracy.

### Theoretical background and clinical relevance of chronotypes

Chronotype represents an individual’s preferred timing for sleep, wakefulness, and peak performance within the 24-hour circadian cycle, reflecting intrinsic differences in the phase and amplitude of endogenous circadian rhythms^1,2^. Originally conceptualized as an organism’s temporal phenotype^12,13^, the construct has evolved to encompass the complex interplay between the endogenous circadian clock located in the suprachiasmatic nucleus and individual behavioral preferences for activity timing^14^. This circadian preference spans a continuum from extreme morningness to extreme eveningness, with most individuals classified as intermediate types who demonstrate moderate temporal preferences^15,16^.

The multifaceted nature of chronotype becomes evident when examining its biological and psychological dimensions^6,17^. From a biological perspective, chronotype correlates with objective markers of circadian timing including dim light melatonin onset (DLMO), core body temperature rhythms, and cortisol secretion patterns^18–20^. These physiological markers complement the psychological aspects, which capture subjective preferences for optimal performance timing and preferred sleep-wake schedules^21^. This dual nature underscores the importance of chronotype as a bridge between biological rhythms and behavioral manifestations^22^. The clinical significance of chronotype research has become increasingly apparent, especially with evening chronotype emerging as a transdiagnostic risk factor across multiple psychiatric conditions. Meta-analytic evidence reveals consistent associations with mood disorders, anxiety symptoms, substance use severity, attentional difficulties, and maladaptive behaviors such as aggression^5,23–25^. Recent large-scale studies demonstrate that 11 out of 13 psychiatric traits are associated with evening chronotype, ranging from depression and social anxiety to obsessive-compulsive disorder and delusional ideation, while only mania shows associations with morning chronotype^26^. Particularly compelling is the longitudinal evidence demonstrating that especially evening chronotype not only precedes but predicts the onset of substance use disorder, depression, and anxiety, thus positioning chronotype assessment as a potential tool for early identification and prevention^27–30^. Beyond its role as a risk factor, chronotype profoundly influences treatment outcomes and therapeutic responses. Evening chronotypes demonstrate differential medication responses, including reduced efficacy to antidepressants and variable responses to lithium therapy in bipolar disorder^4,31,32^. Meta-analyses indicate significantly greater evening chronotype orientation in schizophrenia patients compared to healthy controls, with important implications for symptom severity and treatment planning^33–35^. Conversely, morningness even appears to confer a range of protective effects, again highlighting the profound impact of chronotype on mental health outcomes^36–39^.

Moreover, the relevance of chronotype extends throughout the lifespan, serving as a critical moderator of health behaviors and cognitive performance. Among adolescent populations, chronotype shows both direct and interaction effects with environmental risk factors, contributing to the clustering of maladaptive outcomes^40,41^. In adults, evidence linking chronotype to cognitive decline has been mixed; however, recent longitudinal data offer a more refined perspective. In a 10-year follow-up, evening chronotypes among highly educated adults exhibited more pronounced declines in executive functioning, partly mediated by sleep quality and health behaviors^42^. These results suggest that circadian misalignment may contribute to cognitive vulnerability across adulthood. In conclusion, all these findings have far-reaching consequences for clinical assessment timing, educational scheduling, and workplace optimization, underscoring the necessity of incorporating chronobiological considerations into comprehensive treatment planning, public health interventions, and work-related factors^28,43,44^.

###  Current challenges in chronotype assessment

Considering how chronotype is assessed, there are various instruments available that overlap only to some extent^45^. The *Munich Chronotype Questionnaire*^21^ and the MEQ^6,46^ remain among the most widely utilized chronotype assessment tools, with many of the tools overall offering a classification into seven, five, or three distinct types. Comprehensive validation studies demonstrate robust correlations between MEQ-derived measures and objective circadian markers^20,46^. Studies comparing MEQ scores to DLMO, the gold standard for circadian timing, shows substantial correlations: in a combined regression model including the MEQ, the sleep-corrected midpoint of sleep on free days (MSFsc) from the Munich Chronotype Questionnaire, and age, the two questionnaires showed comparable predictive contributions to DLMO, with the overall model explaining 60% of DLMO variance^19^. This evidence establishes that the MEQ as a composite instrument captures meaningful variance in underlying circadian physiology. However, such validation work is concerned with the aggregate behavior of the instrument rather than with the predictive structure among its constituent items. These are complementary but distinct analytical objectives, and how individual items contribute to chronotype classification has received comparatively little attention, a question that becomes especially pertinent given how the composite score itself is constructed.

Research reveals critical limitations in this approach: Horne and Östberg^6^ assigned different weights to individual items when creating the composite MEQ score yet did not provide a theoretical or empirical rationale for this differential weighting. Subsequent studies have reported low inter-item correlations, suggesting that the assumption of equal contribution, or the validity of the original weighting scheme, may be fundamentally problematic^7,47^. The reduced MEQ (rMEQ) attempts to address some of these limitations by using five items specifically targeting morning activity^48^, but concerns about its factor structure remain unresolved^28^. However, studies increasingly recognize chronotype as a multidimensional construct rather than a unitary trait^41^. A cross-cultural study by Panjeh et al.^9^ employed exploratory factor analysis across four samples (*N*= 3,457) from the UK and Brazil, suggesting that the MEQ might measures three distinguishable constructs rather than a unidimensional chronotype: (1) morning recovery and alertness (involving items 1,3,4,5,7,9,13,19); (2) evening sleepiness and pre-sleep feelings (encompassing items 2,10,12); and (3) peak time of cognitive arousal represented only by item 11. In contrast, sophisticated item-level prediction research has established robust empirical foundations for understanding assessment structures beyond traditional composite scoring^49–51^.

Driving chronotype assessment towards item-level prediction seems promising for two reasons. First, and despite some recent factor-analytic findings, chronotype is not necessarily a latent construct in the traditional sense. While Jordan et al.^52^ conceptualize chronotype within item response theory frameworks as having an underlying latent dimension, the behavioral manifestations of chronotype reflect directly observable circadian preferences rather than abstract psychological constructs. Zou et al.^8^ emphasize that chronotype represents individual variation in the preferred timing of the sleep-wake cycle, with clear physiological correlates including body temperature rhythms and hormone secretion patterns. This manifests behaviorally through specific temporal preferences that can be directly assessed, distinguishing chronotype from truly latent variables like life satisfaction that require inference through multiple indirect indicators. Second, it must be noted that acceptable psychometric properties as assessed by factorial approaches do not necessarily equal predictive utility. Even though chronotype questionnaires usually demonstrate acceptable reliability^45^, the assumption of equal item weighting fails to account for differential predictive power of individual items, potentially diluting the instruments’ clinical utility and ecological validity. This also relates to the various chronotypes themselves. As mentioned, chronotype is traditionally categorized into three to seven distinct types, with both definite and moderate morning and evening, as well as intermediate types. However, there have likewise been different assessment approaches in the literature. For example, some studies operationalized chronotype as either continuous or discrete, or simply combined both for comparison^53,54^. A reasonable number of studies also applied a three-class chronotype categorization with morning, intermediate, and evening types^8,41,55,56^.

Yet, despite the strong biological basis of chronotype, high correlations among measures suggest that fine-grained distinctions between questionnaires may not represent distinct biological or behavioral variations^18,57,58^. Evidence from cardiovascular health research demonstrates meaningful differences primarily between broader morning-evening categories rather than within moderate-definite subcategories, with evening chronotypes showing > 2-fold higher odds of poor cardiovascular health, smoking, and dietary guideline violations compared to morning types^56^. In other words, the emerging evidence on chronotype-health associations suggests that the most robust behavioral and physiological differences tend to occur at the level of broad morning versus evening categories rather than among finer subtypes. This supports the clinical and theoretical relevance of using a merged chronotype classification^28,38,59,60^. Evidence for this category consolidation comes from large-scale epidemiological studies. The landmark study of Roenneberg et al.^16^ including approximately 55,000 individuals demonstrated that chronotype follows a near-normal distribution with the majority clustering in intermediate ranges and a slight over-representation of later chronotypes. Such natural clustering might suggest that the in-between categories, especially in the broader chronotype schemes, may reflect transitional states rather than distinct phenotypes, supporting the consolidation of these into more precise and more clinically meaningful groupings. Age-related chronotype changes further support such a simplified categorization. Research demonstrates that chronotype variability is greatest during adolescence and young adulthood and decreases substantially with age, suggesting that broader categories with more in-between chronotypes may be inappropriate across the lifespan^61^. As a general conclusion, a three-category system of morning, intermediate, and evening types maintains the essential distinction between morning and evening preferences while acknowledging the large intermediate population that lacks strong directional preference. Such a three-type classification might be most suitable for screening purposes like in epidemiology or general clinical research and practice.

### Machine learning classification in chronotype research

The field of psychological assessment is undergoing a paradigm shift from traditional psychometric approaches to predictive machine learning methodologies (see e.g^62–64^.), fundamentally altering how we conceptualize and measure psychological constructs, including chronotypes. This transformation represents a move from theory-driven measurement models to data-driven predictive frameworks that prioritize individual-level prediction accuracy over population-level parameter estimation^10,65,66^.

Traditional psychometric approaches, exemplified by Item Response Theory applied in chronotype scale validation, model the probability of item responses as a function of an underlying latent trait. These approaches typically assume unidimensionality and local independence, with the item-characteristic curves capturing the relationship between the latent trait and response probabilities. However, these may not reflect or capture the complexity of real-world psychological phenomena^67^. Furthermore, conventional methods frequently suffer from overfitting, whereby the model encodes variation unique to the sample rather than genuine underlying relationships, ultimately reducing its generalizability to new data^68^. Machine learning approaches fundamentally differ by prioritizing predictive accuracy and the ability to capture complex, non-linear relationships between variables without requiring a priori assumptions about underlying data structures. Recent comparative studies demonstrate that machine learning methods achieve superior prediction performance compared to traditional item response theory approaches, particularly when modeling individual response patterns rather than population-level estimates^69,70^. This advantage is particularly relevant for chronotype assessment, where individual circadian preferences involve complex interactions between biological, environmental, and behavioral factors that linear models cannot adequately capture. While conventional chronotype measures like the MEQ and MCTQ rely on linear scoring, machine learning approaches can model the complex, non-linear relationships underlying circadian preferences, revealing multidimensional relationships without requiring assumptions about underlying structures.

Correspondingly, machine learning’s primary advantage lies in its ability to capture non-linear relationships and complex interaction effects. Non-linear relationships are ubiquitous in psychological data, where the relationship between measured variables and underlying constructs often follows complex, non-monotonic patterns^71^. Chronotype assessment exemplifies these complexities, where the relationship between individual questionnaire items and overall circadian preference may involve threshold effects, where responses only become predictive above or below certain values of, for example, a Likert-scale. Additionally, interaction effects between items may be crucial in chronotype assessment. Preferences for morning activities may be more predictive of morningness when combined with specific sleep timing preferences. Machine learning algorithms can accommodate these complexities through their flexible functional forms. For example, promising progress has been made to substantiate item response theory with machine learning techniques like neural networks^72^. But for accurate predictions, they usually require immense sample sizes^73^. However, machine learning techniques can likewise be applied for data analysis.

### Rationale and study objectives

Contemporary chronotype assessment methodologies predominantly rely on composite scoring approaches that assume equal item weighting, potentially obscuring differential predictive utilities of individual behavioral indicators – i.e., the individual item-level. This methodological limitation creates a substantial gap between psychometric validity and practical predictive utility required for efficient chronotype classification in clinical and research contexts. To address this limitation, our study pursues three interconnected objectives that bridge theoretical understanding with clinical application. First, we aim to establish a comprehensive machine learning framework for chronotype assessment optimization that can serve as a methodological template for similar psychometric optimization challenges. Second, we seek to identify item-level predictive utility hierarchies, specifically examining how individual items of the German version of the MEQ (DMEQ) contribute to accurate classification across morning, neutral, and evening chronotypes. Third, we intend to examine chronotype-specific predictive patterns and behavioral thresholds that may reveal underlying structural heterogeneity in chronotype assessment which is often not captured by traditional composite scoring methods. Beyond informing chronotype theory, this item-level perspective also provides empirical foundations for principled instrument design. Our analysis yields quantitative information about which items carry the most predictive content and which are comparatively redundant. This allows researchers and clinicians to make informed trade-offs between assessment depth and assessment burden, with explicit knowledge of what is gained or lost at each level. Summarizing, we operationalize the aims of this study according to the following research questions:

- RQ1: Which DMEQ items best predict whether someone is a morning, evening, or neutral chronotype, and does each type depend on different items?
- RQ2: What novel insights about chronotype assessment emerge from machine learning approaches, and to which extent do they correspond to existing psychometric analyses of DMEQ items?
- RQ3: What response patterns separate morning, neutral, and evening types, and what do these patterns reveal about the differences between the chronotypes?

## Methods

### Sample description

Study method and results are reported following the Strengthening the Reporting of Observational Studies in Epidemiology (STROBE) Statement for cohort and cross-sectional studies^74^. The analysis was based on data from the first wave of the Dortmund Vital Study (DVS: ClinicalTrials.gov NCT05155397). The DVS is an interdisciplinary longitudinal study on the sources of interindividual differences in physical, metabolic, immunological, and cognitive functioning throughout working life. The present study used data of the baseline testing, collected between 2016 and 2021. Participants were adults between the ages of 20 and 70, with age distributed almost evenly across the decades. Participants were recruited locally and were generally healthy, with older participants often taking typical medications such as blood pressure medication. The Vital study sample is considered representative in terms of age distribution, genetics, depressive symptoms, cognitive parameters, and occupation. However, the proportion of female participants and those with a university degree is higher than in the general German population. The sample and further details such as eligibility criteria, methods, and procedures are described in detail in the study protocol^75^. Notably, the DVS is considered representative in terms of age distribution, genetics, depressive symptoms, cognitive parameters, and of the distribution of occupations in the German population. Therefore, we can assume that the sample is also representative for chronotype.

The study sample consisted of 612 participants (Table 1). The mean age of the total sample was 43.6 years (SD = 14.6, range 20–70), with morning types being older on average (M = 47.7, SD = 13.2) and evening types being younger (M = 37.1, SD = 13.1). Sex distribution was balanced across chronotype groups. In terms of marital status, divorced participants were slightly more common, particularly among neutral and evening types. Most participants were non-smokers, although smoking was more prevalent among evening types (30%) compared to morning (6%) and neutral types (17%). Mean DMEQ scores differed clearly between groups, with highest values among morning types (M = 63.7, SD = 3.0) and lowest among evening types (M = 37.0, SD = 2.8), reflecting the expected chronotype distinctions. In this study, the full German version of the MEQ^46^ was used. The study was approved by the Institutional Ethics Committee of the Leibniz Research Centre for Working Environment and Human Factors (ID A93-1).

**Table 1:** Sociodemographic Characteristics of the Study Sample.

Variable	Total Sample	Morning Types	Neutral Types	Evening Types
N	612	225	328	68
N after SMOTE application	984	328	328	328
Age years (M ± SD, range)	43.6 ± 14.6 (20–70)	47.7 ± 13.2 (20–70)	41.6 ± 14.7 (20–70)	37.1 ± 13.1 (22–70)
Sex, N (%)				
Male	38	40	38	37
Female	62	60	62	63
Marital status, N (%)				
Single	7.5	8.7	6.6	5.1
Married	37	44.2	32.2	32.2
Divorced	44	39.0	50.9	50.8
Widowed	8	4.7	9.7	10.2
Steady relationship	2	3.5	0.6	1.7
Smoking, N (%)				
Yes	15	6	17	30
No	85	94	83	70
DMEQ score (M ± SD, range)	54 ± 11.0 (23–79)	63.7 ± 3.0 (59–69)	50.5 ± 4.4 (42–58)	37.0 ± 2.8 (31–41)

*SMOTE* Synthetic Minority Over-sampling Technique, *DMEQ* German Version of the Morningness–Eveningness Questionnaire.

However, in the original data the chronotype classes were strongly unbalanced. For example, only eight individuals identified as definite evening types. This pronounced imbalance necessitated strategic methodological decisions. Following non-significant results of variance analysis with sex and age across chronotypes, the original five-category classification scheme was consolidated into three classes: Morning types (*n* = 225, 36.2%), Neutral type (*n* = 328, 52.8%), and Evening types (*n* = 68, 10.9%). Consistent with the existing literature, this consolidation strategy preserves meaningful biological and behavioral distinctions while approximating population prevalence. To ensure robust results, sample sizes within the classes needed to be balanced further. Since this is a known issue when applying machine learning techniques, we used the Synthetic Minority Oversampling Technique (SMOTE)^77^. SMOTE is a synthetic Up-sampling technique. It addresses inherent class imbalances through generation of synthetic samples for underrepresented classes, especially the evening chronotypes in this case. SMOTE’s algorithm identifies k-nearest neighbors and creates synthetic examples along variable space connections between existing observations. Thereby, it produces balanced training sets based on the majority class size (i.e. *N* = 328 for neutral types in this case) without simply replicating the data. This approach maintains within-class variance while preventing majority class bias. Ultimately, this resulted in a dataset with *N* = 328 cases for each, morning, neutral, and evening chronotypes, ensuring robust results.

### Missing data treatment

Data quality evaluation revealed only minimal missing data patterns, with approximately 2% missing responses distributed randomly across participants and questionnaire items, a pattern consistent with Missing Completely At Random (MCAR) mechanisms^78^. This favorable missingness pattern justified the implementation of K-Nearest Neighbor (KNN) imputation^79^ with k = 10 iterations, selected for its demonstrated effectiveness with Likert-scale data and ability to preserve local distributional properties. This imputation strategy maintains item response distributions while avoiding the introduction of a systematic bias. Additional checks included outlier detection, response pattern analysis, and internal consistency validation to ensure robust analytical foundations.

### Analytical approach

Machine learning requires careful analytical strategy and specific performance metrics. Since these approaches may be less familiar than traditional statistics, we provide a detailed explanation of our methods below, also because a major focus of this paper is introducing a novel methodological perspective for chronotype assessment. We first describe the chronotype distribution in our dataset, then explain our algorithm selection and cross-validation procedures. Finally, we present the variable importance measures and performance metrics used to identify influential items and evaluate classification accuracy.

#### Algorithm selection and classification task

Another key question is which algorithm should be used. Extreme gradient boosting (XGBoost)^80^ can be applied for both complex analysis and generating new hypotheses^81^. XGBoost is a powerful algorithm well suited for capturing nonlinear relationships in tabular data that can be applied without making assumptions about the functional form of the relationships between variables - the DMEQ items in this case. Moreover, XGBoost can be combined with post-hoc interpretability methods such as SHapley Additive exPlanations (SHAP values)^82^, which offer a game-theoretic framework for quantifying and visualizing the marginal contribution of each input variable to individual predictions. This approach bridges the gap between predictive accuracy and interpretability, enabling both global and individual-level information^83^. SHAP analysis is suited to reveal which specific questionnaire items or combinations of responses are most predictive of chronotype classification of individual participants, representing a significant advance by providing insight into individual response patterns.

Algorithms like XGBoost can be used, for example, to calculate regressions or classification tasks. Classification refers to a type of machine learning task in which the goal is to assign participants to predefined categories based on patterns in the input data. In the context of chronotype assessment, this means predicting whether individuals are best described as, for example, a morning, evening, or intermediate chronotype based on their questionnaire responses. A classification approach is particularly promising for chronotype assessment because the construct itself is operationalized in terms of discrete categories rather than continuous latent traits. A classifier can learn complex decision boundaries that map response patterns directly onto chronotype categories, potentially increasing accuracy and sensitivity. This allows for a data-driven alternative to rule-based scoring systems, especially in cases where the relationship between items and chronotype is heterogeneous across individuals or subpopulations.

#### Cross-validation and robustness testing

A stratified 10-fold cross-validation provided rigorous assessment of model generalizability while maintaining chronotype proportions across training and validation partitions. This k-fold approach, where k = 10 represents an empirically validated balance between bias and variance in performance estimation enables comprehensive evaluation across diverse data subsets. Each iteration utilized nine folds for training with the remaining fold reserved for unbiased performance assessment, rotating systematically to ensure that all observations contributed to both training and validation phases. Hyperparameter optimization was conducted through automated grid search integrated within the cross-validation framework, systematically evaluating XGBoost parameters. The optimal hyperparameter combination was selected based on cross-validated accuracy performance, with SMOTE sampling applied during each fold to address chronotype class imbalance. This nested approach ensured that hyperparameter selection remained unbiased by test data while maintaining robust performance estimation.

#### Variable importance methods and performance metrics

Overall model performance is first assessed through classification accuracy, reflecting how well the algorithm classified individuals into their original chronotype categories based on response behavior. This is evaluated by the just mentioned cross-validation testing the model’s reliability by repeatedly training it on different portions of the data and testing its predictions on the remaining portions^84^. Accuracy measures how often the model correctly identifies someone’s chronotype out of all predictions made. Sensitivity indicates how well the model detects each specific chronotype when it is actually present, while specificity measures how well it avoids false identifications. The Kappa statistic accounts for agreement that might occur by chance alone, with values above 0.80 indicating strong agreement between predicted and actual chronotypes. The F1-score combines both precision (how many predicted chronotypes were correct) and recall (how many actual chronotypes were detected) into a single balanced measure, with values closer to 1.0 indicating better performance. The AUC (Area Under the Curve) represents the model’s ability to distinguish between different chronotypes across all possible decision thresholds, with values approaching 1.0 indicating nearly perfect discrimination. Collectively, these metrics assess whether the questionnaire data provide sufficient discriminative information to reliably separate morning, intermediate, and evening chronotypes.

Concerning variable importance, machine learning metrics differ fundamentally from traditional statistical approaches such as beta weights in ordinary least squares regression, which measure linear relationships within specific model assumptions. Machine learning importance measures can be categorized into two distinct types based on their analytical focus and methodological approach. First, predictive accuracy measures as exemplified by permutation importance, as used in this study, evaluate the relevance of a variable through systematic variable removal or shuffling^85^. This approach quantifies importance by measuring accuracy degradation when individual variables (i.e. the DMEQ items in this case) are eliminated, providing model-agnostic assessment of true predictive contribution regardless of underlying algorithms^86^. The magnitude of accuracy decrease directly indicates variable importance across the entire dataset, offering global-level insights into feature utility. Second, individual-level attribution methods, represented by SHAP values, provide explanations by calculating each variable’s marginal contribution to an outcome. Unlike the first category, SHAP values quantify how much each DMEQ item pushes individual predictions toward or away from specific chronotype classifications, enabling case-by-case interpretation of model decisions.

The fundamental distinction between both measures lies in the analytical scope: permutation importance reveals which variables matter most for overall model performance, while SHAP values explain the contribution of each variable to individual participant predictions. For example, permutation importance might show that a specific item is the most important predictor overall, while SHAP values would show that Likert-scaled responses of “4” on a 0–6 scale increase the probability of being classified as a Morning type by 0.3 units. However, the same item might decrease another participant’s Morning type probability based on different response pattern. This makes SHAP values analogous to examining residuals or individual case diagnostics in traditional regression, but with the added advantage of revealing how each predictor contributes to both individual and aggregate classifications, rather than merely identifying outliers or poorly fitted cases.

Lastly, we present Partial Dependence Plots. These provide a way to visualize how responses to individual questionnaire items relate to predicted chronotype classifications, while holding other items’ distributions constant. Unlike traditional linear methods, these plots can reveal non-linear patterns, thresholds, and inflection points in the data. In this study, they highlight critical score ranges where small changes in item responses strongly influenced chronotype predictions. For example, certain items might show S-shaped curves for morning types, inverted U-shapes for neutral types, or rapid declines for evening types. These patterns help identifying response ranges most informative for distinguishing chronotypes, supporting both theoretical interpretation and the practical determination of cut-points.

## Results

### Overall classification performance

Cross-validation analysis using stratified 10-fold validation demonstrated robust model performance as displayed in Table 2: accuracy = 94.52% (SD = 2.19%), κ = 0.9046 (SD = 0.0369), mean F1-score = 0.9271, and mean AUC = 0.9869. Class-specific performance showed balanced accuracy across all chronotypes as displayed in Table 3: Morning Types (99.87%), Neutral Type (99.34%), and Evening Types (97.79%), with the minority Evening Types class achieving 95.59% sensitivity despite representing only 10.9% of the sample. This demonstrates that under application of SMOTE, the classifier was able to reliably identify individuals across the full chronotype spectrum with high precision and sensitivity.

**Table 2:** Cross-Validation Classification Performance.

Metric	Mean	Standard Deviation
Accuracy	94.52%	2.19%
Kappa	0.9046	0.0369
Mean F1 Score	0.9271	-
Mean AUC	0.9869	-

**Table 3:** Class-Specific Performance Metrics.

Chronotype	Sensitivity	Specificity	Balanced Accuracy
Morning Types	100.0%	99.75%	99.87%
Neutral Type	99.70%	98.98%	99.34%
Evening Types	95.59%	100.0%	97.79%

### Permutation importance

**Fig. 1:**
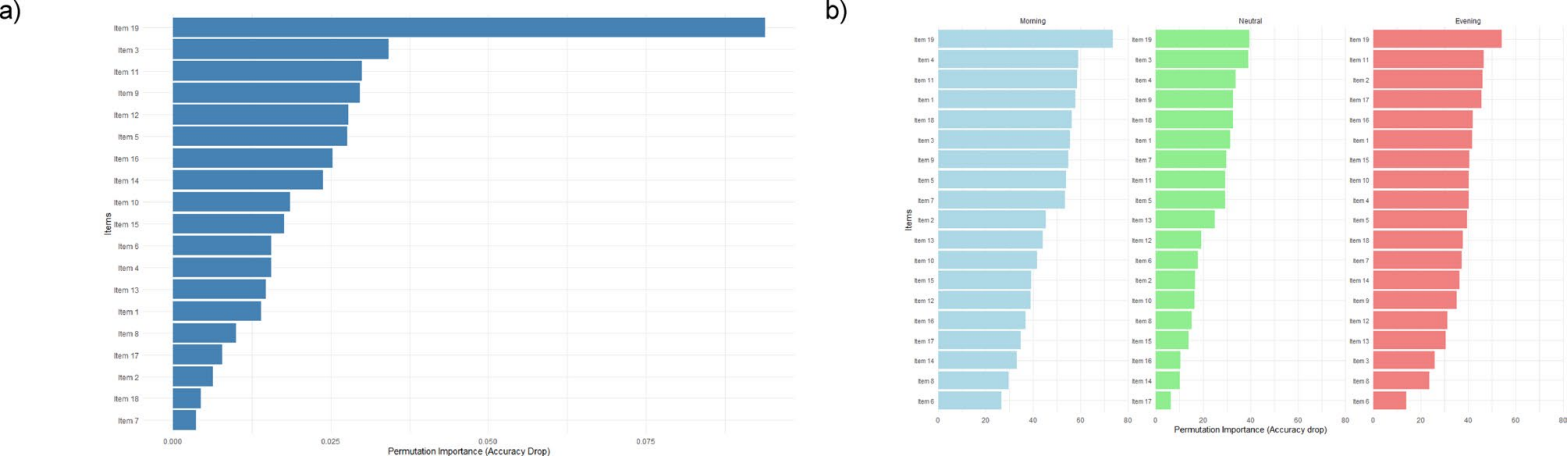
Item-Specific Predictive Accuracy (a) Across the Full Model and (b) for Each Chronotype.

Figure 1 shows how much each (D)MEQ item contributes to the model’s predictive accuracy (a) across the full model and (b) for each chronotype. Item 19 (“Which chronotype do you think you are?”) showed the highest feature importance across chronotypes, with model accuracy decreasing by 10.85% upon its removal, approximately three times higher than that of Item 3, which ranked second. Generally, there is an increasing drop-off after the top six items. The relevance of Item 19 is replicated in chronotype-specific accuracy, but with varying strength. Morning types showed the strongest association with Item 19, whereas intermediate types showed the weakest association. Different secondary predictors emerge for each chronotype, indicating distinct patterns. For example, Items 19, 4, 11, 1, and 18 are important for model accuracy of morning types, with a decreasing accuracy for the following items. Regarding intermediate types, these are Items 19, 3, 4, 9, and 18. For evening types, these are 19, 11, 2, 17 and 16.

### SHAP-based importance

**Fig. 2:**
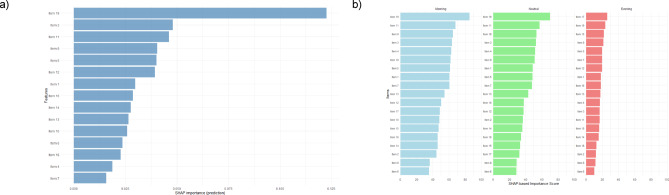
Item-Specific Predictive Contribution (a) Across the Full Model and (b) for Each Chronotype.

The plots in Fig. 2 illustrate individual-level predictive contributions based on SHAP values, showing again (a) overall importance across the full model and (b) chronotype-specific importance. Item 19 again shows the highest predictive contribution overall and across all chronotypes. The full model identifies Items 19, 3, 11, 9, 5 and 12 as having the greatest predictive contributions. As with permutation importance, predictive relevance decreases after the top six items. Across chronotypes, there are class-specific patterns demonstrating specific hierarchies. For Morning types, the most influential items are 19 and 11, followed by items 9, 3, 4, and items from 18, 5, 1 and 7 being of similar relevance. Neutral types also show the highest sensitivity to Items 19 and 11, followed by the same items as with morning types but in a different sequence. Evening types are most influenced by Items 17 and 19, with the other items following a gradual decline in predictive power. Moreover, the relevance of items for predicting evening types is less strong compared to morning and neutral types.

**Fig. 3:**
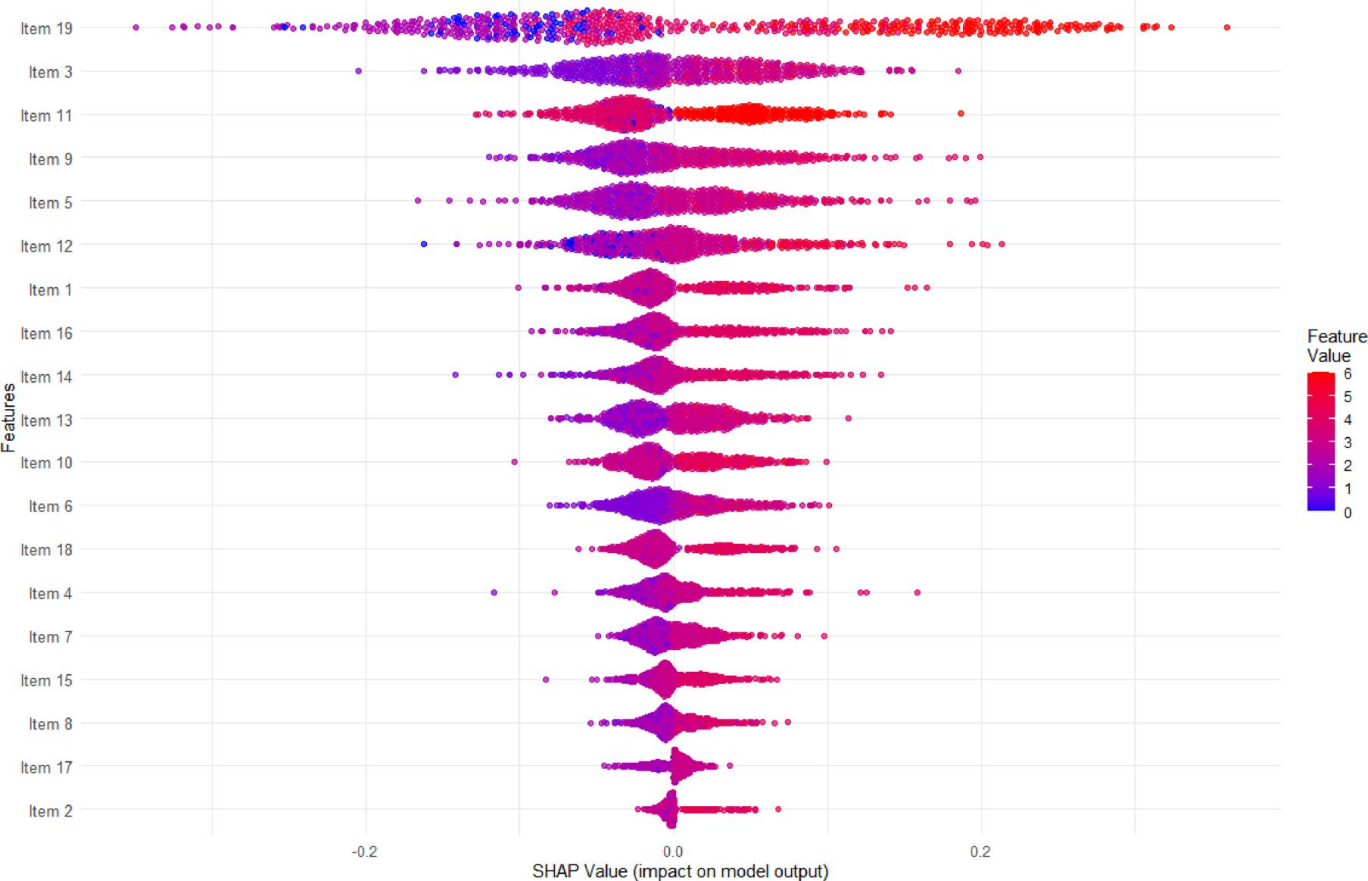
Bee Swarm Plot: SHAP Item Impact on Chronotype Prediction Across all Types. The observed SHAP value range reflects the range of predictive influence individual DMEQ items exert within this specific dataset and model configuration.

The Bee-Swarm plot in Fig. 3 shows how each (D)MEQ item contributes to individual chronotype predictions using SHAP values. Each dot represents one individual’s response, with colors indicating their answer on the 0–6 scale (blue = low scores, red = high scores). Item 19 again shows the strongest impact and discriminative power across all chronotypes, meaning that this item can strongly push predictions toward different chronotypes depending on individual responses. Items with clustered SHAP values near zero (such as those at the bottom of the plot) have minimal predictive influence, whereas items with broader value distributions (Items 19, 16, and 9) contribute more substantially to distinguishing between chronotypes.

Several items display bimodal clustering patterns (e.g. Item 19, 11), where responses tend to have either strong positive or negative effects with few neutral contributions, suggesting threshold effects where moderate responses have minimal impact while extreme responses drive classification decisions. The color gradients reveal a monotonic relationship between response value and SHAP contribution on the (D)MEQ’s 0–6 Likert scale: high item scores (red, values 5–6) produce the most positive SHAP values, pushing individuals toward morning classification; moderate scores (purple/pink, values 3–4) cluster near the center of the SHAP distribution with small contributions in either direction; and low scores (blue, values 0–1) produce the most negative SHAP values, pushing toward evening classification. Predictive strength therefore increases toward both extremes of the response scale rather than concentrating at a single pole. Predictive strength therefore increases toward both extremes of the response scale rather than concentrating at a single pole. Looking especially at items 3 and 11, they show clear color separation, indicating that higher item values consistently push predictions towards a specific chronotype, meaning they show a substantial discriminatory power. The wide spread of these items furthermore indicate these items to matter a lot for individual predictions. Items with mixed colors on both sides indicate non-linear and interaction effects between items. Only Item 3 seems to be mostly independent from such effects. While Items 19, 3 and 11 capture most discriminatory power, items 9, 5, and 12 add nuance and capture additional variance.

Table 4 lists the (D)MEQ items together with their full question wording, the corresponding mean SHAP value per item, and the resulting rank in classification prediction. The hierarchy mirrors the importance pattern shown across Fig. 2a and b, as well as Fig. 3. The table is intended as a reference complementing the figures, allowing to map item numbers directly to question content.

**Table 4:** MEQ items with corresponding question wording and mean SHAP importance.

Item	Question	Mean SHAP	Rank
1	What time would you get up if you were entirely free to plan your day?	0.030	7
2	What time would you go to bed if you were entirely free to plan your evening?	0.010	19
3	If there is a specific time at which you have to get up in the morning, to what extent do you depend on being woken up by an alarm clock?	0.048	2
4	How easy do you find it to get up in the morning (when you are not woken up unexpectedly)?	0.019	14
5	How alert do you feel during the first half hour after you wake up in the morning?	0.040	5
6	How hungry do you feel during the first half-hour after you wake up in the morning?	0.024	12
7	During the first half-hour after you wake up in the morning, how tired do you feel?	0.015	15
8	If you have no commitments the next day, what time would you go to bed compared to your usual bedtime?	0.012	17
9	You have decided to engage in some physical exercise. A friend suggests that you do this for one hour twice a week and the best time for him is between 7:00–8:00 am. Bearing in mind nothing but your own internal “clock”, how do you think you would perform?	0.041	4
10	At what time of day do you feel you become tired as a result of need for sleep?	0.025	11
11	You want to be at your peak performance for a test that you know is going to be mentally exhausting and will last for two hours. You are entirely free to plan your day. Considering only your own internal “clock”, which ONE of the four testing times would you choose?	0.046	3
12	If you got into bed at 11:00 PM, how tired would you be?	0.040	6
13	For some reason you have gone to bed several hours later than usual, but there is no need to get up at any particular time the next morning. Which ONE of the following are you most likely to do?	0.026	10
14	One night you have to remain awake between 4:00–6:00 AM in order to carry out a night watch. You have no commitments the next day. Which ONE of the alternatives will suite you best?	0.028	9
15	You have to do two hours of hard physical work. You are entirely free to plan your day and considering only your own internal “clock” which ONE of the following time would you choose?	0.011	18
16	You have decided to engage in hard physical exercise. A friend suggests that you do this for one hour twice a week and the best time for him is between 10:00–11:00 PM. Bearing in mind nothing else but your own internal “clock” how well do you think you would perform?	0.029	8
17	Suppose that you can choose your own work hours. Assume that you worked a FIVE hour day (including breaks) and that your job was interesting and paid by results). Which FIVE CONSECUTIVE HOURS would you select?	0.013	16
18	At what time of the day do you think that you reach your “feeling best” peak?	0.022	13
19	One hears about “morning” and “evening” types of people. Which ONE of these types do you consider yourself to be?	0.122	1

Mean SHAP importance corresponds to the values shown in Fig. 2a. Rank refers to the ordering of items by Mean SHAP importance. Item wording reproduced from the English version of the MEQ (Horne & Östberg, 1976); the German version (DMEQ; Griefhahn et al., 2001) was used in the analyses.

### Partial dependence plots

**Fig. 4:**
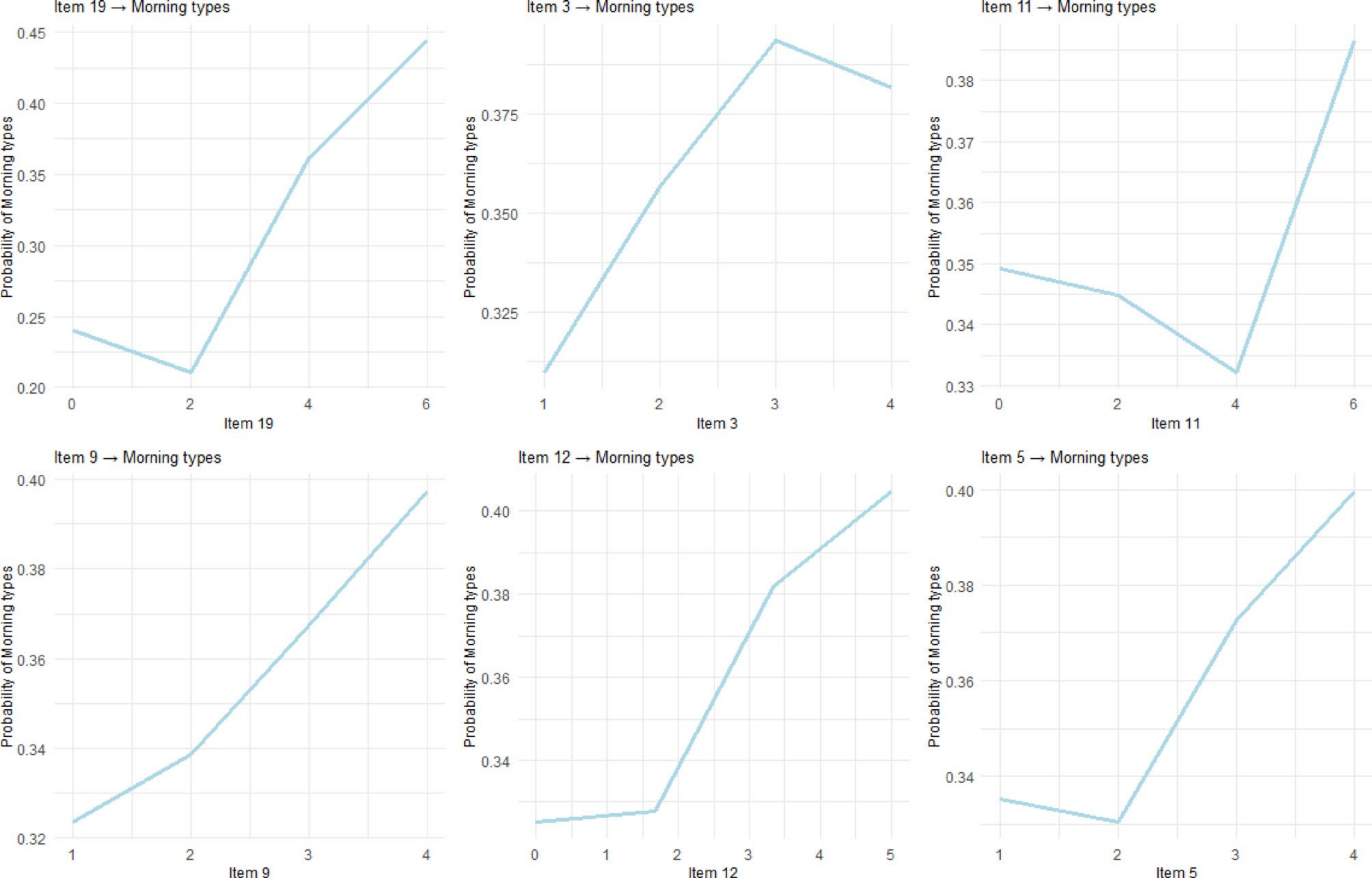
Partial Dependance Plots for the Top Five Items Predicting Morning Types. The x-axis scale varies by item based on the actual response range of the (D)MEQ on a 0-5 or 0-6 Likert scale.

**Fig. 5:**
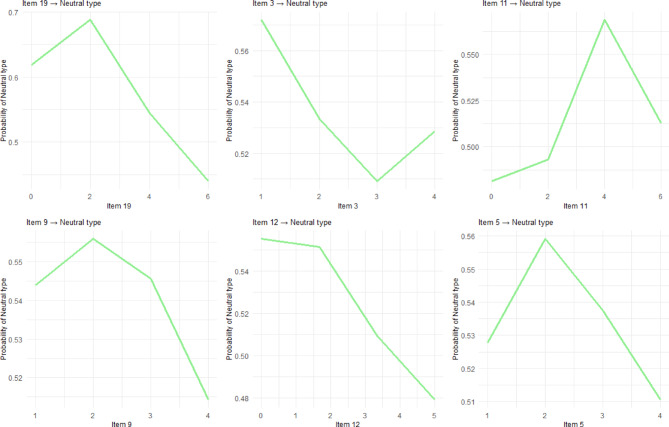
Partial Dependance Plots for the Top Five Items Predicting Neutral Types. The x-axis scale varies by item based on the actual response range of the (D)MEQ on a 0-5 or 0-6 Likert scale.

**Fig. 6:**
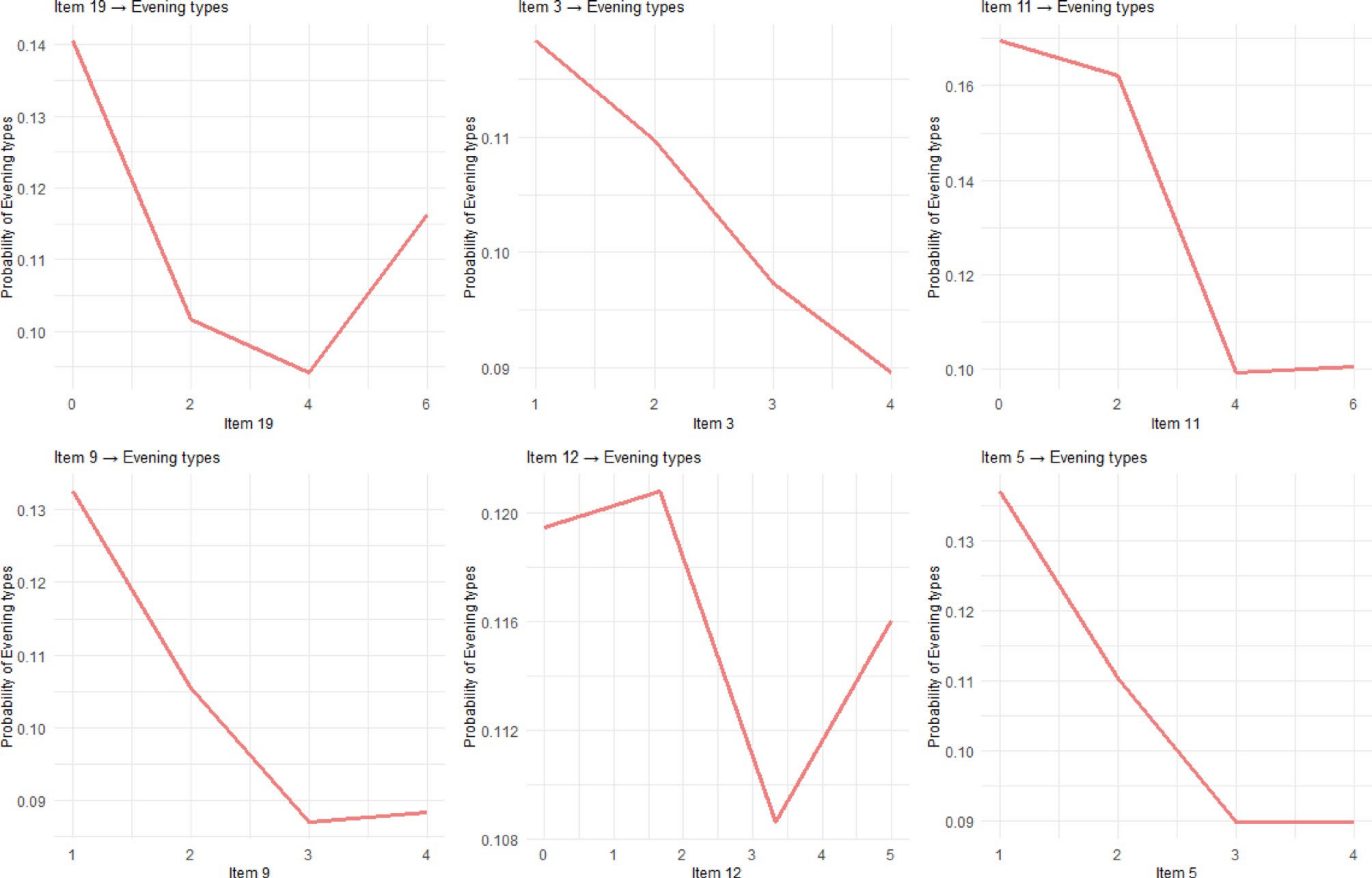
Partial Dependance Plots for the Top Five Items Predicting Evening Types. The x-axis scale varies by item based on the actual response range of the (D)MEQ on a 0-5 or 0-6 Likert scale.

Partial dependence plots for each chronotype (Figs. 4, 5 and 6) reveal distinct non-linear response patterns across (D)MEQ items. For example, Item 19 demonstrates a characteristic threshold effect for Morning type classification: responses from 0 to 2 on the Likert scale produce minimal probability changes, indicating limited predictive value at lower scale levels. A critical inflection occurs between scores 2–4, followed by steep, approximately linear increases from 4 to 6, creating the characteristic S-shaped curve typical of threshold-based biological and behavioral phenomena.

## Discussion

The present study employed machine learning methods to investigate and optimize chronotype assessment using the German Morningness–Eveningness Questionnaire, addressing fundamental questions regarding item-level predictive utility and the underlying structure of chronotype measurement. By applying extreme gradient boosting, we achieved a classification accuracy of 94.52% across chronotypes while simultaneously demonstrating substantial heterogeneity in the predictive contributions of individual questionnaire items. Our findings challenge core assumptions of traditional psychometric approaches and provide an empirical basis for reconceptualizing chronotype assessment within broader theoretical and clinical frameworks.

### Item 19: a metacognitive self-assessment

Among all questionnaire items, the most striking result emerged for Item 19, whose exceptional predictive relevance highlights the central role of metacognitive self-assessment in chronotype determination. This single question *“Which chronotype do you think you are?”* demonstrated threefold greater importance than any other item, and its removal from the model led to a 10.85% reduction in classification accuracy. By contrast, items positioned at the lower end of the importance hierarchy accounted for less than 1% of classification accuracy. The prominence of Item 19 indicates that individuals’ direct judgments about their own chronotype encapsulate a remarkably accurate summary of their chronotype. This finding aligns with Turco et al.^87^, who demonstrated that a single self-classification question with a wording comparable to the MEQ’s Item 19 reliably assesses diurnal preference when validated against complete questionnaires, subjective sleepiness patterns, and actigraphic measures. Extending this line of evidence, Aronna-Palacios and Diaz-Morales^88^ further demonstrated the cross-cultural validity of chronotype self-assessment in both Mexican and Spanish adolescent populations, suggesting that this capacity for accurate self-classification may be a robust and generalizable feature across diverse contexts.

The implications of these findings extend beyond the straightforward validation of a single questionnaire item. From a psychometric perspective, Item 19 represents more than an efficient proxy for longer scales; it appears to capture a fundamental dimension of chronotype-related self-awareness. Panjeh et al.^9^ reported that this item loads strongly on their Factor 1, representing the efficiency of dissipation of sleep pressure, reflecting how individuals experience morning recovery processes. This observation suggests that metacognitive awareness of chronotype may be rooted primarily in subjective experiences of morning adaptation rather than in evening preferences or peak performance timings. Consistent with this interpretation, our SHAP analysis revealed an unusually wide value range for Item 19, demonstrating that responses to this question exert an outsized influence on model predictions in either direction. In practical terms, the item functions as a pivotal “classification lever,” shifting predicted chronotype toward morningness or eveningness depending on the response. High responses on Item 19 strongly predict morning chronotype, while low responses predict the opposite. This item alone captures substantial discriminatory power, offering value for clinical research and practice.

Viewed in a broader theoretical context, the strong predictive utility of Item 19 supports recent theoretical perspectives suggesting that chronotype encompasses more than biological circadian preferences^8^. It suggests that individuals possess accurate self-knowledge about their chronotype derived from lived experience. Importantly, chronotype undergoes systematic changes across the lifespan, shifting toward eveningness during adolescence and reverting toward morningness in later adulthood^61,89^, with longitudinal evidence demonstrating continued transitions even in middle to late adulthood^90^. Yet individuals appear capable of accurately recognizing their current chronotype position at any developmental stage. When answering which chronotype they think they are, individuals draw on years of direct observation of their own sleep-wake preferences, energy patterns, and daily functioning. This accumulated self-knowledge seems to be reliable. The finding validates that chronotype self-assessment captures genuine biological preferences effectively, supporting the development of abbreviated assessment tools that rely on individuals’ established understanding of their own circadian patterns.

### Reconceptualizing questionnaire structure

Traditional MEQ scoring assumes an equal contribution of all 19 items. Our analysis demonstrates that this assumption may be fundamentally flawed. The extreme variation in predictive utility across items reveals that certain items contribute disproportionately to classification, while others play only a negligible role. When mapping our variable importance hierarchy onto the Panjeh et al.^9^ three-factor structure, some interesting discrepancies emerge. Their Factor 1 (morning recovery/dissipation of sleep pressure) encompasses Items 1, 3, 4, 5, 7, 9, 13, and 19. While several of these items, most notably Items 3, 4, 9, and 19, also appear among our top predictors, their relative contributions vary significantly. This divergence between factor loadings and predictive importance highlights a critical epistemological distinction: psychometric validity does not equate to predictive utility. Factor loadings reflect the extent to which items cluster conceptually, whereas predictive importance measures how effectively items classify individuals. Items may therefore load strongly on a latent factor while contributing little to actual classification accuracy. While Urban and Bauer^91^ demonstrated that deep learning approaches can improve item factor analysis through better handling of non-linear relationships and computational efficiency, they remain within the factor analytic framework. The distinction between psychometric validity and predictive utility becomes most apparent when comparing traditional factor-based approaches with classification-focused machine learning methods like ours. Zang et al.^72^ similarly showed that neural network approaches can capture complex patterns in psychometric data that linear models miss. In contexts such as chronotype measurement, where the goal is practical classification rather than latent trait estimation, machine learning approaches reveal which items effectively drive predictive performance beyond their factorial associations.

### Dynamic and heterogeneous mechanisms of chronotype classification

So far, our analysis has revealed that chronotype classification is shaped both by non-linear response patterns within individual items and by distinct mechanisms that differentiate morning, evening, and neutral types. The partial dependence plots furthermore emphasized that several items, most notably Item 19, follow sigmoid trajectories that create meaningful behavioral boundaries. For Item 19, a critical inflection between scores 2–4 on the Likert scale marks the point at which morningness probability increases sharply. This suggests the presence of a chronotype threshold rather than a gradual continuum. These non-linear dynamics not only refine but also contextualize traditional MEQ scoring. Directional assumptions remain broadly valid, since higher scores on morning-preference items still predict morningness, lower scores predict eveningness. But machine learning reveals more complex shapes of these relationships. Some items exhibited steep threshold effects, others gradual transitions, and several contribute little to classification. This refined view situates chronotype as a construct shaped by functional inflection points rather than uniform linear gradients.

Most importantly, understanding these dynamics also specifically illuminates why classification mechanisms differ across chronotypes. Morning types, for instance, rely heavily on items tied to early-day functioning (Items 1, 4, and 11). Their predictive profiles suggest that chronotype identity here is anchored in the dissipation of sleep pressure and recovery efficiency, which are processes consistent with strong homeostatic (Process S) influence^92,93^. Evening types, by contrast, draw more strongly on items capturing delayed phase and evening alertness (Items 2, 11, and 17), highlighting a greater role for circadian (Process C) dynamics^19,61^. Neutral types, in contrast, display flatter partial dependence curves and more distributed pattern of item importance, consistent with weaker circadian amplitude rather than a discrete phenotype. Evidence of reduced circadian gene expression among individuals with intermediate chronotypes supports this interpretation, suggesting that “neutral” may reflect attenuated rhythmicity rather than a distinct category^8,94^. While this item-specific sensitivity might not seem surprising, it is, again, the response behavior revealed by non-linear modeling that proves decisive.

Taken together, these findings highlight that classification differences across chronotypes are not merely reflections of the circadian phase position. Chronotypes seem to emerge from the interplay of threshold effects, non-linear item dynamics, and asymmetric reliance on circadian versus homeostatic mechanisms across groups. This perspective helps to explain why developmental shifts in chronotype often appear abrupt^61,90^ and why psychiatric risk associated with eveningness^8,23^ may be linked to categorical transitions rather than continuous gradients. Beyond the mechanistic interpretation, the chronotype-specific structure of item importance is itself a substantive finding: composite scoring approaches treat all items as equally informative for all chronotypes, but our results show that the information carried by each item varies systematically with the class to be identified. The identification of different chronotypes effectively relies on different items, which is a regularity that is structurally invisible under uniform-weight scoring and which has direct implications for how chronotype assessment can be conceptualized.

### Validation of ultra-brief assessment strategies

The exceptional predictive utility of Item 19 presents immediate and transformative opportunities for ultra-brief chronotype assessment. With appropriate validation against full questionnaires and objective circadian markers, this single self-assessment question could serve as a rapid screening tool in resource-constrained contexts such as primary care consultations or large-scale epidemiological surveys. The potential impact is considerable, given growing evidence that chronotype is linked to cardiovascular risk, metabolic dysfunction, and psychiatric vulnerability^8,56^. For a more comprehensive yet still abbreviated assessment, our analyses identify Items 19, 3, 11, 9, 5, and 12 as a balanced six-item combination for general chronotype classification. This empirically derived set achieves robust classification across all chronotypes while reducing assessment burden by more than 70%. The choice of six items was anchored on the conventional length of existing abbreviated instruments rather than on an empirically forced cut-off in the importance distribution, which shows a gradual decline below the prominent gap separating Item 19 from the remaining items. A slightly longer or shorter set would be equally defensible on the predictive evidence alone.

Notably, this selection differs from the reduced MEQ^48^, which includes items 1, 7, 10, 18, and 19. While both recognize Item 19’s importance, our machine learning approach reveals that questions like Items 3 (morning freshness), 9 (morning alertness), or 11 (peak performance time) provide superior predictive utility compared to several rMEQ items. This six-item combination could be immediately implemented in clinical and research settings, offering a practical balance between brevity and accuracy for chronotype assessment across diverse populations.

Other abbreviated chronotype instruments provide additional points of comparison. The five-item Morningness–Eveningness Scale for Children (MESC^95^) was developed specifically for adolescent populations, while the ultra-short Munich ChronoType Questionnaire^96^ uses six items focusing on sleep timing. The single-item chronotyping (SIC) method^97^ employs visual charts for self-assessment that correspond to Item 19’s approach, as both require participants to identify which chronotype they think they are. Additionally, the Morningness–Eveningness Stability Scale (MESSi^98^) incorporates amplitude assessment alongside phase preference in 15 items.

In addition, the empirically-derived thresholds identified through partial dependence analysis could form the foundation for providing clinical cutoffs superior to traditional percentile-based categories. These thresholds enable evidence-based decision-making regarding intervention timing and intensity. For instance, individuals scoring in the 2–4 transition zone on Item 19 may benefit from preventive interventions before full evening chronotype develops, potentially interrupting the pathway to psychiatric vulnerability documented in longitudinal studies^90,99^.

### Implementation framework and reproducibility

To facilitate widespread adoption, we provide a comprehensive open-source R tutorial in the Supplementary Material, demonstrating the complete analytical pipeline. This framework enables researchers to validate findings across diverse populations while adapting the methodology for other psychological assessments. The tutorial includes automated functions for handling class imbalances, hyperparameter optimization specific to questionnaire data, and generation of interpretable visualizations that communicate findings to non-technical audiences.

Recent applications of machine learning for psychological assessment demonstrate the broader potential of this approach. Studies employing SHAP-based item analysis have successfully optimized measures of worry, anxiety, and depression, consistently identifying opportunities for questionnaire refinement while maintaining or improving predictive validity^100^. Our framework extends these applications by providing a complete workflow from raw data to clinical implementation, addressing a critical gap in translating machine learning insights into practical assessment tools.

### Limitations and future directions

Several limitations warrant consideration when interpreting these findings. First, while our model achieves high classification accuracy against questionnaire-derived categories, we did not include validation against objective biological markers of circadian timing such as DLMO, actigraphy, or core body temperature rhythms. Our findings therefore identify items with high predictive utility for chronotype classification but cannot speak directly to how well an abbreviated form built from these items would track underlying physiology. Future prospective studies should test whether such an abbreviated form maintains physiological correlations, and whether items demonstrating high predictive utility in our analysis also relate to objective physiological indicators^46^.

Second, despite SMOTE’s effectiveness in addressing class imbalance, our original sample contained limited evening type representation. While synthetic sample generation preserves within-class variance, it does not create genuinely new information. This limitation becomes relevant given that particularly extreme evening types, although representing a small population share, demonstrate qualitatively different patterns of circadian gene expression and psychiatric vulnerability compared to moderate evening types. Relatedly, our reported item-level predictive hierarchies are estimated against this three-category classification target, and we expect the core hierarchy to remain broadly stable also under a finer-grained target, since the moderate and definite subdivisions represent positions along a continuous chronotype distribution centered on the three principal categories^16^. This remains a theoretical expectation, however, and warrants direct examination in future work with samples adequate for five-category analysis. This limitation goes hand in hand with the third one.

Thirrd, s limitation goes hand in hand with the third one. Although the Dortmund Vital Study is a registered cohort study, the sample is German speaking and was furthermore relatively small compared to other studies with much larger datasets, potentially capturing population-based distributions of chronotypes much better. However, the study is considered representative for several biological indicators on which circadian preferences ground. For such further analysis and retesting of our approach, we provided the R tutorial in this manuscript.

Fourth, the cross-sectional design prevents assessment of temporal stability in predictive patterns. The generalizability of these findings across the lifespan remains uncertain, as chronotype and its behavioral correlates change with age. Future longitudinal research should examine whether item-level predictive patterns remain stable or vary across developmental stages.

Fifth and finally, current chronotype instruments including the DMEQ primarily capture sleep-related preferences while overlooking non-sleep circadian manifestations. Recent theoretical models emphasize chronotype as a multidimensional construct encompassing temperature rhythms, hormone patterns, cognitive performance fluctuations, and social temporal preferences^14^. Our analysis remains necessarily limited to dimensions captured by existing items, potentially missing critical aspects of circadian phenotype that future comprehensive assessments should incorporate. The study’s strength lies in bridging predictive machine learning with clinical trans-lation. Given chronotype’s documented associations with psychiatric risk, treatment outcomes, and health behaviors across the lifespan, efficient and accurate assessment tools have immediate practical value for screening in clinical practice, large-scale epidemiological research, and workplace optimization. Our findings directly address persistent challenges in chronotype measurement: the tension between com-prehensive assessment and practical brevity, and the gap between psychometric validity and predictive utility.

## Conclusion

This investigation demonstrates how machine learning approaches can reconceptualize chronotype assessment by revealing item-level predictive heterogeneity obscured by traditional psychometric methods. Most notably, Item 19 (“Which chronotype do you think you are?“) showed the highest discriminative power, demonstrating that individuals’ metacognitive self-assessment captures essential information about their circadian timing and daily functioning. Combined with chronotype-specific importance patterns and non-linear response relationships, this item-level perspective challenges assumptions underlying composite scoring and replaces conceptual intuition about which items ought to matter with a quantitative hierarchy of how much each one actually does. This hierarchy is itself class-specific: identifying morning, neutral, and evening types relies on different items - a pattern uniform-weight scoring cannot capture. On this empirical basis, a six-item version achieves robust classification performance across chronotypes, illustrating how the same evidence can be drawn on to develop efficient, transparent, and potentially personalized chronotype assessment strategies. The broader contribution is a framework that bridges predictive performance and theoretical understanding, advancing both scientific knowledge and clinical practice.

## Supplementary Information

Below is the link to the electronic supplementary material.

Supplementary Material 1

## Data Availability

The data supporting the findings of the study will be made available upon reasonable request.

## Declarations

### Data transparency

The data supporting the findings of the study are publicly available upon reasonable request.
